# Association between steatotic liver disease and microvascular complications in individuals with type 2 diabetes: a cohort study in the UK Biobank

**DOI:** 10.3389/fendo.2025.1554798

**Published:** 2025-05-28

**Authors:** Longfu Liu, Qiqi You, Wenxiang Yu, Aaron M. Lett, Yucen Wu, Jingjing Zeng, Menglin Fan, Bo Chen, Wan Fu, Shaoyong Xu

**Affiliations:** ^1^ Department of Endocrinology, Xiangyang Central Hospital, Affiliated Hospital of Hubei University of Arts and Science, Xiangyang, Hubei, China; ^2^ Center for Clinical Evidence-Based and Translational Medicine, Xiangyang Central Hospital, Affiliated Hospital of Hubei University of Arts and Science, Xiangyang, Hubei, China; ^3^ Department of Metabolism, Digestion and Reproduction, Imperial College London, London, United Kingdom

**Keywords:** steatotic liver disease, type 2 diabetes, microvascular complications, cohort, glycemic control

## Abstract

**Background:**

Cross-sectional studies have revealed that steatotic liver disease (SLD) is associated with prevalent diabetic microvascular complications, but longitudinal evidence in large samples is insufficient. We aimed to prospectively investigate the association between SLDs and the risk of microvascular complications in patients with type 2 diabetes (T2D), and to explore whether glycemic control played a mediating role in this association.

**Methods:**

The population-based cohort, which was based on the UK Biobank study, included 25,630 T2D patients at baseline. SLD was defined as a fatty liver index ≥ 60. A glycated hemoglobin level ≥ 7% (53 mmol/mol) was considered poor glycemic control. The primary outcome was total incident diabetic microvascular complications, defined as the first occurrence of diabetic nephropathy, diabetic neuropathy, and/or diabetic retinopathy. The cox proportional hazard regression model was used to calculate hazard ratios (HRs) and 95% confidence intervals (CIs) for diabetic microvascular complications. Mediation analysis was applied to explore whether the association between SLDs and diabetic microvascular complications was mediated by glycemic control.

**Results:**

The mean age of the study participants was 59.6 years, and 58.1% of them were males. During a median follow-up period of 12.1 years, 5,171 participants were diagnosed with microvascular complications. Compared with non-SLD participants, SLD participants had a HR of 1.15 (95% CI: 1.04, 1.27) for total microvascular complications, a HR of 1.20 (95% CI: 1.06, 1.35) for diabetic nephropathy, a HR of 1.05 (95% CI: 0.91, 1.21) for diabetic retinopathy, and a HR of 1.46 (95% CI: 1.15, 1.86) for diabetic neuropathy. The results of the mediation analysis revealed that the mediating proportion of glycemic control in the association between the SLD group and total diabetic microvascular complications was 22.5% (95% CI: 10.4%, 91.0%).

**Conclusions:**

SLD was associated with an increased risk of microvascular complications, especially diabetic nephropathy and diabetic neuropathy, in T2D patients. Glycemic control partially mediated the association between SLDs and diabetic microvascular complications.

## Introduction

Type 2 diabetes (T2D) is a common chronic disease with increasing prevalence worldwide and poses a major public health challenge (1). T2D patients may experience a variety of chronic complications, and microvascular complications are of particular concern because they greatly reduce patients’ quality of life and can lead to disability or premature death (2). While glycemic control is a known influencing factor, evidence also suggests that lipid metabolism substantially impacts the development of chronic diabetic complications (3).

Steatotic liver disease (SLD) is characterized by abnormal accumulation of fat in the liver, and is chosen as an overarching term to cover the various causes of steatosis (such as alcoholic and metabolic associated steatosis) according to the latest delphi consensus statement (4). SLD is common in T2D patients; for example, the prevalence of nonalcoholic fatty liver disease is estimated to be as high as 65% (5). Furthermore, in T2D patients, SLD is associated with an increased risk of macrovascular complications and mortality (6, 7). However, whether SLD increases the risk of diabetic microvascular complications in this population has not yet been systematically investigated. To date, studies have predominantly explored the associations between SLDs and individual microvascular complications (such as diabetic nephropathy, diabetic retinopathy, or diabetic neuropathy) through cross-sectional or case-control designs of small samples (8–13), which may be underpowered in terms of statistical efficiency.

The liver plays a critical role in maintaining glucose homeostasis, and abnormal increases in gluconeogenesis and impaired glycogen metabolism are observed in the SLD (14). Considering that microvascular complications are driven mainly by chronic hyperglycemia (15), we hypothesized that SLDs in T2D patients may influence the occurrence of microvascular complications by affecting glycemic control. Therefore, we aimed to investigate whether the SLD was associated with an increased risk of microvascular complications in T2D patients, and to explore whether glycemic control played a mediating role in this association in the UK Biobank cohort.

## Materials and methods

### Study design and population

Our data were sourced from the UK Biobank, which is a large cohort that included approximately 500,000 middle-aged and elderly volunteers at baseline between March 2006 and July 2010 (16). The follow-up of participants involved linkage with hospital admission records from England, Scotland, and Wales, as well as the national death register. Further details about the database can be found elsewhere (17).

A total of 28,078 participants aged between 40 and 72 years at the baseline survey were identified with T2D by using the International Classification of Diseases, 10th edition (ICD-10) code, blood glucose and glycated haemoglobin levels (18). Participants who met the following criteria were excluded: those with missing data for SLD (*n* = 1,746); and those with diabetic microvascular diseases at baseline (*n* = 702). Finally, 25,630 participants were included in the main analysis (**Supplementary Figure 1**).

### Ascertainment of the baseline SLD

With no liver biopsy data available and only a small number of T2D patients having proton density fat fraction data in the UK Biobank (19), our study adopted a well-established index, the fatty liver index (FLI), to evaluate hepatic steatosis. In brief, FLI is a simple and noninvasive predictor of hepatic steatosis, with an accuracy of 0.84 (95% confidence interval (CI): 0.81, 0.87). The FLI is derived through a specific formula that incorporates body mass index (BMI), waist circumference, triglyceride, and gamma-glutamyl transpeptidase, and is calculated as:


$$\begin{array}{c} \text{FLI}={\text{e}}^{\wedge }{\;}^{(0.953\;*\text{ loge }\left(\text{triglycerides}\right)\;}{\;}^{+\;0.139\;*\text{ body mass index }}\\ {\;}^{+\;0.718\;*\text{ loge }\left(\text{gamma glutamyltransferase}\right)}{\;}^{+\;0.053\;*\text{ waist circumference }-\;15.745)}\\ /(1+e^{\wedge \;(0.953\;*\text{ loge }\left(\text{triglycerides}\right)\;+\;0.139\;*\text{ body mass index}}\\ {\;}^{+\;0.718\;*\text{ loge }\left(\text{gamma glutamyltransferase}\right)\;+\;0.053\;*\text{ waist circumference }-\;15.745)})\;*\;100 \end{array}$$

(20).

The FLI score ranges from 0 to 100, and a FLI score ≥ 60 is defined as SLD, which has been widely used in large cohort studies (19, 21).

### Ascertainment of outcome

The primary outcome was total incident diabetic microvascular complications, which was defined as the first occurrence of diabetic nephropathy, diabetic neuropathy, and/or diabetic retinopathy. The secondary outcomes included the incidence of each subtype of diabetic microvascular complications. The occurrence of each outcome was identified through linkage to the National Health Service in England, the Information and Statistics Division in Scotland, and the Secure Anonymised Information Linkage in Wales. The diagnosis was documented via the following ICD-10 codes: diabetic nephropathy (E11.2, E14.2, N08.3, N18.0, N18.1, N18.2, N18.3, N18.4, N18.5, N18.8, N18.9); diabetic retinopathy: (E11.3, E14.3, H28.0, H36.0); and diabetic neuropathy (E11.4, E14.4, G59.0, G62.9, G63.2, G99.0) (22, 23).

### Ascertainment of covariates

The participants’ baseline information was obtained via the UK Biobank via touch-screen questionnaires, physical measurements or linkages with other authorities, etc. Information on age, sex and the Townsend deprivation index was acquired from local NHS primary care trust registries. The Townsend deprivation index is a measure of material deprivation, with a higher score indicating greater poverty (24).

Additional sociodemographic information (i.e. ethnicity and education level) and lifestyle information were collected through touch-screen questionnaires. Smoking status was classified into never smoking, former smoking, current smoking, and unwilling to answer. Alcohol intake frequency was stratified into seven groups, ranging from never drinking to daily or almost daily drinking, plus those unwilling to answer. Physical activity was categorized into high, medium, and low groups on the basis of International Physical Activity Questionnaire guidelines (25). Medication information was obtained with the following question: “Do you regularly take any of the following medications? (you can select more than one answer)”. Diabetes duration was the time span from the diagnosis of T2D to engagement in the baseline survey.

Height, weight and waist circumference were measured by trained nurses. BMI was calculated as BMI (kg/m^2^) = weight (kg)/height^2^ (m^2^) and was divided into three groups: normal weight (BMI < 25 kg/m^2^), overweight (25 ≤ BMI < 30 kg/m^2^) and obesity (BMI ≥ 30 kg/m^2^), according to the World Health Organization standard (26). Blood pressure (mmHg) was recorded as the average of two readings from the Omron device. The platelet count (10^^9^ cells/l) was the number of thrombocytes derived from the platelet histogram. Blood glucose (mmol/l), lipids (mmol/l) and several enzymes that reflect liver function (u/l) were measured via a Beckman Coulter AU5800. Glycated haemoglobin was measured via a Bio-Rad VARIANT II Turbo, and a value equal to or greater than 7% (53 mmol/mol) was considered poor glycemic control (27).

### Statistical analysis

All analyses were performed using the Stata 17.0 (Stata Corp, College Station, TX, USA) and RStudio (version 2023.09.1) software. A *P* value < 0.05 was considered statistically significant. Continuous variables are presented as means ± standard deviations or medians (interquartile ranges), and categorical variables are presented as numbers (percentages). Differences were assessed using the t-tests, Mann-Whitney U tests and Chi-square tests. The missing covariates were addressed by multiple imputation method (28).

The cox proportional hazard regression model was used to calculate hazard ratios (HRs) and corresponding 95% CIs of diabetic microvascular complications in the SLD group compared with the non-SLD group. The follow-up time was calculated in calendar years from the baseline participation date to the date of outcome or death or September 30, 2021. A Schoenfeld residual test was performed to validate the proportional hazards assumption, and the P-value (> 0.05) indicated no violation of the assumption. We also categorized the FLI score into quartile groups or as a continuous variable (19), and assessed the presence of liver fibrosis to explore the association between the degree of hepatic steatosis and the risk of diabetic microvascular complications. The fibrosis-4 index (FIB-4) was used to define advanced liver fibrosis and was calculated as FIB-4 = (age * aspartate aminotransferase)/(platelet * alanine aminotransferase ^^1/2^). The low cut-off point and high cut-off point of the FIB-4 score were 1.30 and 2.67, respectively (29). In addition, we explored whether the association between the SLD group and microvascular complications in T2D patients was partially mediated by glycemic control through mediation analysis (30).

We investigated the association between the SLD group and the risk of diabetic microvascular complications stratified by age (< 65 years and ≥ 65 years), sex (male and female), ethnicity (White and non-white), diabetes duration (< 1 year, 1-5 years, and > 5 years), and BMI (< 25 kg/m^2^ and ≥ 25 kg/m^2^). Several sensitivity analyses were performed to test the stability of the results. First, we excluded participants whose outcomes occurred within two years after baseline to avoid possible reverse causality. Second, we excluded participants with missing covariates to examine the impact of missing data on the results. Third, we re-analyzed the data by using FLI tripartite classification (FLI ≥ 60, SLD group; 30-59, intermediate group; and FLI < 30, no-SLD group) to reduce potential misclassification bias (6, 20).

## Results

### Participant characteristics

The study involved 25,630 T2D patients with a mean age of 59.6 years, and 58.1% were males. Among them, 18,488 had SLD, accounting for 72.1%. Compared with T2D patients with non-SLD, those with SLD were more likely to be males, have a higher Townsend deprivation Index, be current smokers, and have low levels of physical activity, and they were less likely to have a college or higher education level (*P* < 0.001). T2D patients with SLD also showed a longer duration of diabetes, larger BMI and waist circumference, higher blood pressure, and higher levels of blood glucose, glycated haemoglobin, triglycerides and enzymes that reflect liver function, compared to those with non-SLD (*P* < 0.001) (**Table 1**).

**Table 1:** Baseline characteristics of the study population.

Baseline characteristics^a^	Total population (n = 25,630)	Non-SLD group (n = 7,142)	SLD group (n = 18,488)	*P-*value
Age (year)	59.6 ± 7.1	59.8 ± 7.3	59.5 ± 7.0	0.001
Male, n (%)	14,894 (58.1)	3,082 (43.2)	11,812 (63.9)	<0.001
Ethnicity, n (%)				<0.001
White	22,542 (88.0)	5,975 (83.7)	16,567 (89.6)	
Mixed	161 (0.6)	51 (0.7)	110 (0.6)	
Asian	1,544 (6.0)	640 (9.0)	904 (4.9)	
Black	753 (2.9)	259 (3.6)	494 (2.7)	
Other	557 (2.2)	197 (2.8)	360 (2.0)	
Education, n (%)				<0.001
College/University	6,257 (24.4)	2,219 (31.1)	4,038 (21.8)	
A levels/AS levels	2,364 (9.2)	684 (9.6)	1,680 (9.1)	
O levels/GCSEs	5,013 (19.6)	1,427 (20.0)	3,586 (19.4)	
CSEs	1,179 (4.6)	252 (3.5)	927 (5.0)	
NVQ/HND/HNC	2,093 (8.2)	426 (6.0)	1,667 (9.0)	
Other professional qualifications/None of the above	8,149 (31.8)	1,972 (27.6)	6,177 (33.4)	
Prefer not to answer	575 (2.2)	162 (2.3)	413 (2.2)	
Townsend deprivation index	-1.4 (-3.3, 1.8)	-1.9 (-3.6, 1.0)	-1.2 (-3.2, 2.0)	<0.001
Smoking status, n (%)				<0.001
Never	11,880 (46.4)	3,908 (54.7)	7,972 (43.1)	
Previous	10,644 (41.5)	2,446 (34.3)	8,198 (44.3)	
Current	2,831 (11.1)	717 (10.0)	2,114 (11.4)	
Prefer not to answer	202 (0.8)	51 (0.7)	151 (0.8)	
Alcohol intake frequency, n (%)				<0.001
Never	3,813 (14.9)	1,056 (14.8)	2,757 (14.9)	
Special occasions only	4,464 (17.4)	1,132 (15.9)	3,332 (18.0)	
One to three times a month	3,109 (12.1)	753 (10.5)	2,356 (12.7)	
Once or twice a week	5,992 (23.4)	1,649 (23.1)	4,343 (23.5)	
Three or four times a week	4,193 (16.4)	1,288 (18.0)	2,905 (15.7)	
Daily or almost daily	3,932 (15.3)	1,227 (17.2)	2,705 (14.6)	
Prefer not to answer	54 (0.2)	17 (0.2)	37 (0.2)	
IPAQ activity group, n (%)				<0.001
Low	5,307 (20.7)	995 (13.9)	4,312 (23.3)	
Moderate	8,041 (31.4)	2,359 (33.0)	5,682 (30.7)	
High	6,507 (25.4)	2,269 (31.8)	4,238 (22.9)	
Other	5,775 (22.5)	1,519 (21.3)	4,256 (23.0)	
Diabetes duration, n (%)				<0.001
< 1 year	16,088 (62.8)	4,860 (68.1)	11,228 (60.7)	
1-5 years	5,064 (19.8)	1,211 (17.0)	3,853 (20.8)	
> 5 years	4,478 (17.5)	1,071 (15.0)	3,407 (18.4)	
Insulin use, n (%)	328 (1.3)	179 (2.5)	149 (0.8)	<0.001
Body mass index (kg/m^2^)	31.1 ± 5.9	25.5 ± 2.8	33.3 ± 5.3	<0.001
Waist circumference (cm)	101.7 ± 14.8	86.1 ± 8.8	107.7 ± 11.9	<0.001
Systolic blood pressure (mmHg)	141.9 ± 17.9	139.2 ± 18.5	143.0 ± 17.6	<0.001
Diastolic blood pressure (mmHg)	82.2 ± 10.1	79.1 ± 9.7	83.5 ± 10.0	<0.001
Platelet count (10^9^/l)	249.7 ± 65.0	252.2 ± 65.7	248.8 ± 64.6	<0.001
Blood glucose (mmol/l)	8.0 ± 3.1	7.7 ± 2.8	8.1 ± 3.2	<0.001
Glycated hemoglobin (mmol/mol)	51.9 ± 15.0	47.5 ± 15.3	53.6 ± 14.5	<0.001
Triglycerides (mmol/l)	2.3 ± 1.3	1.5 ± 0.7	2.6 ± 1.4	<0.001
High density lipoprotein cholesterol (mmol/l)	1.2 ± 0.3	1.4 ± 0.4	1.1 ± 0.3	<0.001
Gamma glutamyltransferase (u/l)	36.7 (24.8, 58.8)	23.4 (17.8, 32.3)	43.8 (30.4, 69.4)	<0.001
Alanine aminotransferase (u/l)	25.4 (18.7, 35.7)	19.5 (15.4, 24.9)	28.5 (21.1, 39.6)	<0.001
Aspartate aminotransferase (u/l)	25.3 (21.1, 31.5)	23.2 (20.0, 27.1)	26.4 (21.8, 33.6)	<0.001

a Continuous variables were expressed as means ± standard deviations or medians (interquartile ranges), and categorical variables were expressed as numbers (percentages).

SLD, steatotic liver disease; IPAQ, International Physical Activity Questionnaire.

### Association of the SLD with diabetic microvascular complications

After a median follow-up of 12.1 years, 5,171 diabetic microvascular complications were observed. Among these patients, 3,258 had diabetic nephropathy, 2,098 had diabetic retinopathy, and 1,033 had diabetic neuropathy. According to the results of the log-rank test, the cumulative incidence of diabetic microvascular complications was significantly higher in the SLD group than in the non-SLD group (*P* < 0.001) (**Supplementary Figure 2**).

**Figure 1:**
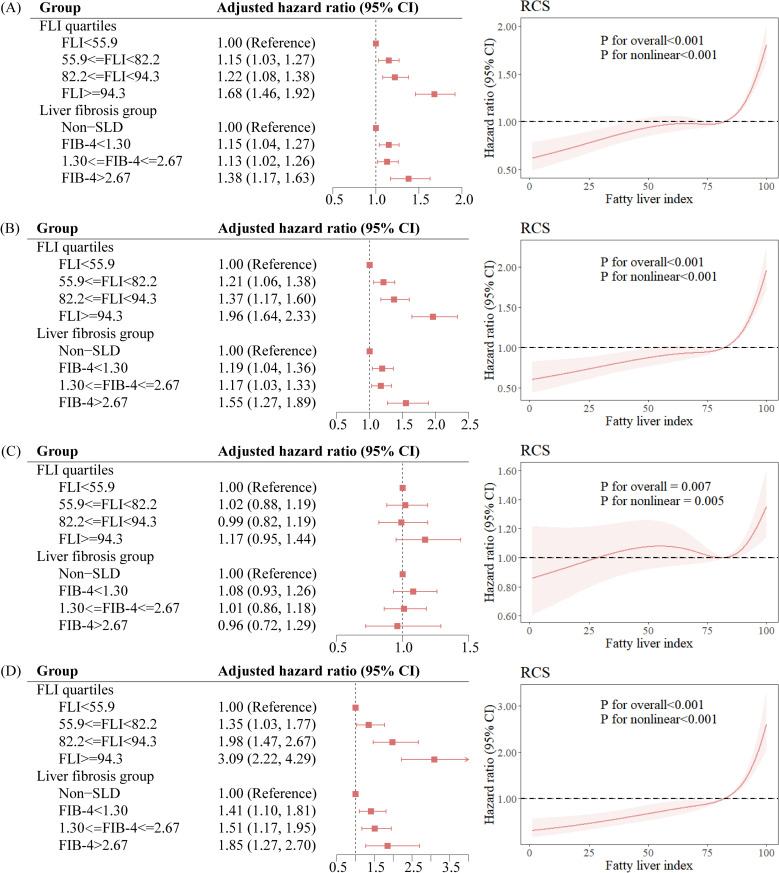
Association between the degree of hepatic steatosis and the risk of diabetic microvascular complications. **(A)** Total microvascular complications, **(B)** Nephropathy, **(C)** Retinopathy, **(D)** Neuropathy. FLI, fatty liver index; SLD, steatotic liver disease; CI, confidence interval; RCS, restricted cubic spline.

Compared with non-SLD participants, SLD participants had a HR of 1.15 (95% CI: 1.04, 1.27) for total microvascular complications, a HR of 1.20 (95% CI: 1.06, 1.35) for diabetic nephropathy, a HR of 1.05 (95% CI: 0.91, 1.21) for diabetic retinopathy, and a HR of 1.46 (95% CI: 1.15, 1.86) for diabetic neuropathy (**Table 2**). Compared with participants in the lowest quartile of FLI, participants in the highest quartile of FLI had a HR of 1.68 (95% CI: 1.46, 1.92) for total microvascular complications. Compared with non-SLD participants, SLD participants with FIB-4 ≥ 2.67 also had a 38% (HR: 1.38, 95% CI: 1.17, 1.63) higher risk of total microvascular complications. The results of the restricted cubic spline curve indicated a significant nonlinear association between FLI scores and the risk of total microvascular complications (*P*_for nonlinear_ < 0.001) (**Figure 1**).

**Table 2:** Association between SLD group and the risk of diabetic microvascular complications.

Group	Events/Participants	Incidence rate (per 1000 person-years)	Hazard ratio (95% confidence interval)	
			Model 0	Model 1
Total microvascular complications				
Non-SLD group	1,013/7,142	12.4 (11.7, 13.2)	1.00 (Reference)	1.00 (Reference)
SLD group	4,158/18,488	20.6 (20.0, 21.2)	1.69 (1.57, 1.81) ***	1.15 (1.04, 1.27) **
Nephropathy				
Non-SLD group	571/7,142	6.8 (6.3, 7.4)	1.00 (Reference)	1.00 (Reference)
SLD group	2,687/18,488	12.9 (12.4, 13.4)	1.91 (1.75, 2.09) ***	1.20 (1.06, 1.35) **
Retinopathy				
Non-SLD group	472/7,142	5.6 (5.2, 6.2)	1.00 (Reference)	1.00 (Reference)
SLD group	1,626/18,488	7.7 (7.3, 8.1)	1.37 (1.24, 1.52) ***	1.05 (0.91, 1.21)
Neuropathy				
Non-SLD group	147/7,142	1.7 (1.5, 2.0)	1.00 (Reference)	1.00 (Reference)
SLD group	886/18,488	4.1 (3.9, 4.4)	2.40 (2.02, 2.86) ***	1.46 (1.15, 1.86) **

Model 0: unadjusted; Model 1: adjusted for age, sex, ethnicity, education level, Townsend deprivation index, smoking status, alcohol intake frequency, physical activity, diabetes duration, insulin use, body mass index, hypertension, triglyceride and high-density lipoprotein cholesterol.

SLD, steatotic liver disease.

*P < 0.05, **P < 0.01, ***P < 0.001.

### Mediation analysis

**Figure 2:**
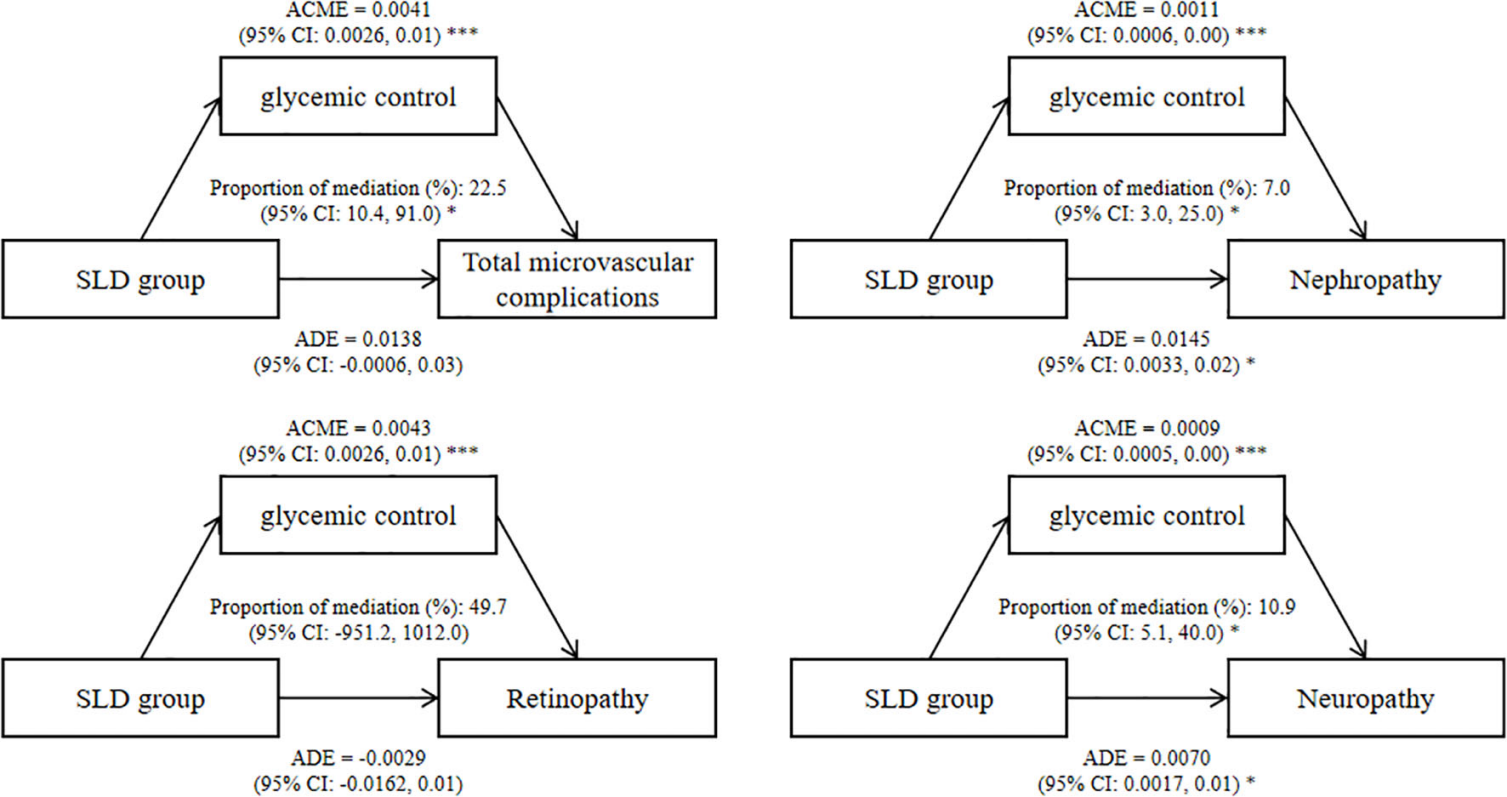
Mediating effects of SLD group on diabetic microvascular complications by glycemic control. Effect and proportion estimations were adjusted for age, sex, ethnicity, education level, Townsend deprivation index, smoking status, alcohol intake frequency, physical activity, diabetes duration, insulin use, body mass index, hypertension, triglyceride and high-density lipoprotein cholesterol. ADE, average direct effects; ACME, average causal mediation effects; CI, confidence interval; SLD, steatotic liver disease. *P < 0.05, ***P < 0.001.

The results of the mediation analysis revealed that glycemic control played a mediating role in the association between the SLD group and diabetic microvascular complications, and the mediating proportion was 22.5% (95% CI: 10.4%, 91.0%) for total microvascular complications (**Figure 2**). The association between the SLD group and glycemic control as well as the association between glycemic control and diabetic microvascular complications was shown in **Supplementary Table 1**.

### Subgroup analysis and sensitivity analysis

Subgroup analysis revealed that in T2D patients who were under 65 years of age (HR: 1.25, 95% CI: 1.09, 1.43), female (HR: 1.24, 95% CI: 1.05, 1.45), White (HR: 1.15, 95% CI: 1.03, 1.28), had a diabetes duration of over 5 years (HR: 1.21, 95% CI: 1.00, 1.45), and were overweight or obese (HR: 1.39, 95% CI: 1.27, 1.53), SLD was significantly associated with an increased risk of total microvascular complications, while no significant association was found in the remaining subgroups (**Supplementary Table 2**). Sensitivity analysis indicated no significant changes in the direction or magnitude of the association between the SLD group and diabetic microvascular complications (**Supplementary Table 3**).

## Discussion

Our study used data of 25,630 T2D patients from the UK Biobank to systematically explore the association between SLD and the risk of diabetic microvascular complications. There were three key findings: Firstly, SLD was associated with a 15% increased risk of total microvascular complications compared to non-SLD, with a significant association with diabetic nephropathy and diabetic neuropathy (*P* < 0.01). Secondly, increased degree of hepatic steatosis, as assessed by the FLI score and liver fibrosis, was associated with increased risks of microvascular complications. Lastly, glycemic control mediated the association between the SLD group and total microvascular complications by 22.5%.

Hepatic steatosis is an important risk factor for macrovascular complications and mortality in T2D patients (6, 31), but its association with microvascular complications is inconsistent, and there are few studies on total microvascular complications (32, 33). One case-control study explored the effect of nonalcoholic fatty liver disease on total diabetic microvascular complications, and did not find a significant association (odds ratio:0.53, 95% CI: 0.11, 2.49) (32). Notably, only 935 T2D patients were included in this study, which may lead to imprecise estimates of effects and lower confidence in the results. Another cohort study found that nonalcoholic fatty liver disease was independently and positively associated with the development of microvascular diseases (adjusted HR: 1.45, 95% CI: 1.28, 1.63) (33), which was consistent with our findings.

In terms of individual microvascular complications, we found that SLD was positively associated with the risk of diabetic nephropathy and diabetic neuropathy. The harmful effects of hepatic steatosis on chronic kidney disease have been extensively studied in the general population (34, 35), and a positive association was also observed in patients with diabetes (13, 36, 37), which was in line with our findings. Additionally, recent evidence highlights the role of cholemic nephropathy, a condition characterized by renal dysfunction secondary to hyperbilirubinemia, in subclinical renal injury. For instance, a study suggested that even acute mild hyperbilirubinemia may induce early renal tubular damage in the absence of alterations of the normal parameters used in clinical practice (38). While our study focused on SLD-associated metabolic disturbances, future research may explore whether elevated bilirubin levels in SLD patients contribute to diabetic nephropathy via cholemic mechanisms. This could further elucidate the hepatic-renal axis in T2D patients and refine risk stratification strategies. The results regarding the association between SLDs and diabetic neuropathy were consistent with the findings of a prospective study conducted in Iran (13) and several cross-sectional studies (10). In this prospective study, the incidence risk of diabetic neuropathy was significantly increased in patients with nonalcoholic fatty liver disease (odds ratio: 1.34, 95% CI: 1.09, 1.64) (13).

Current findings on the association between SLD and diabetic retinopathy are contradictory. Our study did not find a significant association between the two, which is consistent with a prior cohort study (3,123 Iranians) and cross-sectional study (5,963 Americans) reporting non-significant odds ratios of 0.90 (95% CI: 0.71, 1.13) and 0.77 (95% CI: 0.47, 1.26) for nonalcoholic fatty liver disease and diabetic retinopathy, respectively (13, 39). Two cross-sectional studies reported a lower prevalence of diabetic retinopathy in T2D patients with nonalcoholic fatty liver disease (12, 40). These discrepant results may be due to differences in study design, sample size and participants’ ethnicity. The two studies had small sample sizes from Asian countries (929 Koreans and 411 Chinese). In contrast, Targher et al. suggested that nonalcoholic fatty liver disease was associated with an increased prevalence of proliferative/laser-treated retinopathy in T2D patients in Italy (HR: 1.75, 95% CI: 1.10, 3.70), but no significant association was found with non-proliferative retinopathy (HR: 1.19, 95% CI: 0.80, 1.70), suggesting that the type of diabetic retinopathy may also influence the association (11).

Liver fibrosis is a more serious disease state than hepatic steatosis. Several studies have explored its association with diabetic microvascular complications, most of which were consistent with our findings (41–43). For example, Rosa Lombardi et al. reported that significant liver fibrosis was independently associated with the presence of microvascular complications (adjusted odds ratio: 4.20, 95% CI: 1.50, 11.40) (42). However, in the study by Niloofar Deravi et al., liver fibrosis assessed by the FIB-4 was not significantly associated with diabetic microvascular complications (13). This may be because that the reference group in this study was the first tertile of FIB-4, which may include patients with SLD, thus reducing the difference between groups.

Previous literature has shown that patients with SLD have abnormally increased gluconeogenesis and impaired glycogen metabolism (14), which is related to the occurrence of diabetic microvascular complications (44). In our study, we quantified the role that glycemic control played in the association between SLDs and microvascular complications, mediated by 22.5%. This may suggest that T2D patients with hepatic steatosis have worse glycemic control than T2D patients alone, which needs to be considered in the clinical treatment and management of patients with diabetes and hepatic steatosis. The remaining approximately 77% of the effect may be related to inflammation, lipid metabolism and other pathways. Hepatic steatosis is closely associated with chronic low-grade inflammation, characterized by elevated pro-inflammatory cytokines such as TNF-α, IL-6, and C-reactive protein (45). These inflammatory mediators may directly impair endothelial function, promote oxidative stress, and exacerbate insulin resistance, thereby contributing to microvascular damage (46). Furthermore, altered lipid metabolism in SLD, including increased free fatty acid flux, hepatic overproduction of triglyceride-rich lipoproteins, and ectopic lipid deposition in peripheral tissues, may induce lipotoxicity and mitochondrial dysfunction in microvascular cells (47).

### Strengths and limitations

The strengths of this study lie in the utilization of a large population-based sample and a long follow-up period. On the basis of investigating the association between SLDs and diabetic microvascular complications, we further explored the possible mediating effect of glycemic control. However, some limitations should be acknowledged. Firstly, the gold standard for diagnosing hepatic steatosis is liver biopsy, but this is difficult to implement in large epidemiological studies, and there is no available data on liver biopsy in the UK Biobank. Therefore, we utilized a well-established and validated index to assess hepatic steatosis (20). Secondly, despite our efforts to adjust for potential confounders, the possibility of residual confounding may remain. Thirdly, microvascular complications in our study were identified by using ICD-10 codes linked with hospital records. This may result in underdiagnosis and undercoding of microvascular complications, potentially leading to an underestimation of its incidence. Fourthly, the wide confidence interval of the mediating effect may reflect the sample size limitation in mediation analyses and biological variability of this study, which need cautious interpretation and validation in large-scale longitudinal studies. Lastly, the UK Biobank cohort predominantly comprises data from participants across the UK. Caution is needed when extending our results to other ethnic groups, as the demographics may differ.

### Conclusions

SLD was associated with an increased risk of microvascular complications, especially diabetic nephropathy and diabetic neuropathy, in T2D patients. Glycemic control partially mediated the association between SLDs and diabetic microvascular complications. In the course of clinical diagnosis and treatment, it is advisable to routinely screen T2D patients for hepatic steatosis and inform them about the potential increased risk of microvascular complications. Priority should be given to GLP-1 agonists or SGLT2 inhibitors in T2D patients with SLD to treat both hyperglycemia and hepatic steatosis. Future studies may explore other biological pathways mediating the association between SLDs and diabetic microvascular complications to provide a basis for precise interventions in T2D patients.

## Data Availability

The datasets presented in this study can be found in online repositories. The names of the repository/repositories and accession number(s) can be found below: https://www.ukbiobank.ac.uk.
