# Supplementary material for: Association between steatotic liver disease and microvascular complications in individuals with type 2 diabetes: a cohort study in the UK Biobank
Source: Front Endocrinol (Lausanne). 2025 May 28;16:1554798. doi: 10.3389/fendo.2025.1554798 (PMC12151786; doi:10.3389/fendo.2025.1554798)
Supplement: Supplementary file 1 [file DataSheet1.docx]

**List of supplementary materials**

**Supplementary Table 1** Association between the SLD group and glycemic control, as well as association between glycemic control and diabetic microvascular complications

**Supplementary Table 2** Subgroup analysis on the association between SLD group and the risk of diabetic microvascular complications

**Supplementary Table 3** Sensitivity analysis on the association between SLD group and the risk of diabetic microvascular complications

**Supplementary Figure 1** Study flow chart

**Supplementary Figure 2** Cumulative incidence of diabetic microvascular complications

**Supplementary Table 1** Association between the SLD group and glycemic control, as well as association between glycemic control and diabetic microvascular complications

| **Dependent variable** | **Independent variable** | **Odds ratio (95% confidence interval)** | ***P*-value** |
| --- | --- | --- | --- |
| Glycemic control ^a^ | SLD group | 1.29 (1.18, 1.42) | <0.001 |
| Total microvascular complications ^b^ | Glycemic control | 1.70 (1.59, 1.81) | <0.001 |
| Nephropathy ^b^ | Glycemic control | 1.25 (1.15, 1.35) | <0.001 |
| Retinopathy ^b^ | Glycemic control | 2.73 (2.47, 3.01) | <0.001 |
| Neuropathy ^b^ | Glycemic control | 1.80 (1.57, 2.06) | <0.001 |

^a^ Binary logistic regression model with glycemic control as the dependent variable and the SLD group as the independent variable. Adjusted for age, sex, ethnicity, education level, Townsend deprivation index, smoking status, alcohol intake frequency, physical activity, diabetes duration, insulin use, body mass index, hypertension, triglyceride and high density lipoprotein cholesterol

^b^ Binary logistic regression model with diabetic microvascular complications as the dependent variable and glycemic control as the independent variable. Adjusted for age, sex, ethnicity, education level, Townsend deprivation index, smoking status, alcohol intake frequency, physical activity, diabetes duration, insulin use, body mass index, hypertension, triglyceride, high density lipoprotein cholesterol, and SLD group

SLD, steatotic liver disease

**Supplementary Table 2** Subgroup analysis on the association between SLD group and the risk of diabetic microvascular complications

| **Subgroup** | **Hazard ratios (95% confidence intervals)** | | | |
| --- | --- | --- | --- | --- |
|  | **Total microvascular complications** | **Nephropathy** | **Retinopathy** | **Neuropathy** |
| **Age** |  |  |  |  |
| < 65 years | 1.25 (1.09, 1.43) ** | 1.37 (1.14, 1.64) ** | 1.08 (0.89, 1.31) | 1.37 (1.00, 1.89) |
| ≥ 65 years | 1.07 (0.93, 1.23) | 1.06 (0.90, 1.26) | 1.04 (0.83, 1.31) | 1.68 (1.15, 2.44) ** |
| **Sex** |  |  |  |  |
| Male | 1.04 (0.92, 1.17) | 1.13 (0.96, 1.33) | 0.89 (0.74, 1.06) | 1.29 (0.96, 1.73) |
| Female | 1.24 (1.05, 1.45) * | 1.25 (1.02, 1.53) * | 1.23 (0.96, 1.58) | 1.58 (1.01, 2.46) * |
| **Ethnicity** |  |  |  |  |
| White | 1.15 (1.03, 1.28) * | 1.22 (1.06, 1.40) ** | 1.01 (0.85, 1.19) | 1.37 (1.05, 1.78) * |
| Non-white | 1.17 (0.93, 1.48) | 1.03 (0.76, 1.40) | 1.28 (0.94, 1.74) | 2.27 (1.21, 4.26) * |
| **Diabetes duration** |  |  |  |  |
| < 1 year | 1.11 (0.96, 1.27) | 1.15 (0.96, 1.38) | 1.02 (0.84, 1.26) | 1.38 (0.97, 1.97) |
| 1-5 years | 1.12 (0.91, 1.36) | 1.15 (0.90, 1.47) | 0.93 (0.68, 1.28) | 1.42 (0.87, 2.31) |
| > 5 years | 1.21 (1.00, 1.45) * | 1.30 (1.03, 1.64) * | 1.13 (0.85, 1.49) | 1.51 (0.97, 2.36) |
| **Body mass index** |  |  |  |  |
| < 25 kg/m^2^ | 0.75 (0.50, 1.11) | 0.91 (0.55, 1.51) | 0.47 (0.21, 1.03) | 0.45 (0.18, 1.18) |
| ≥ 25 kg/m^2^ | 1.39 (1.27, 1.53) *** | 1.51 (1.34, 1.70) *** | 1.21 (1.06, 1.38) ** | 1.99 (1.55, 2.54) *** |

Hazards ratios were calculated by adjusting for age, ethnicity, education level, Townsend deprivation index, smoking status, alcohol intake frequency, physical activity, diabetes duration, insulin use, body mass index, hypertension, triglyceride and high density lipoprotein cholesterol

SLD, steatotic liver disease

* *P* < 0.05, ** *P* < 0.01, *** *P* < 0.001

**Supplementary Table 3** Sensitivity analysis on the association between SLD group and the risk of diabetic microvascular complications

|  | **Hazard ratios (95% confidence intervals)** | | | |
| --- | --- | --- | --- | --- |
|  | **Total microvascular complications** | **Nephropathy** | **Retinopathy** | **Neuropathy** |
| **Excluding participants with outcome diagnosed within two years after baseline (n = 25,009)** | | | | |
| Non-SLD group | 1.00 (Reference) | 1.00 (Reference) | 1.00 (Reference) | 1.00 (Reference) |
| SLD group | 1.13 (1.02, 1.25) * | 1.18 (1.04, 1.34) * | 1.03 (0.88, 1.20) | 1.40 (1.09, 1.82) * |
| **Excluding participants with missing covariates (n = 23,966)** | | | | |
| Non-SLD group | 1.00 (Reference) | 1.00 (Reference) | 1.00 (Reference) | 1.00 (Reference) |
| SLD group | 1.12 (1.02, 1.24) * | 1.17 (1.03, 1.33) * | 1.03 (0.89, 1.20) | 1.45 (1.13, 1.87) ** |
| **Using tripartite classification of fatty liver index (n = 25,630)** | | | | |
| No-SLD group | 1.00 (Reference) | 1.00 (Reference) | 1.00 (Reference) | 1.00 (Reference) |
| Intermediate group | 1.10 (0.95, 1.28) | 1.04 (0.85, 1.26) | 1.10 (0.88, 1.37) | 1.25 (0.85, 1.84) |
| SLD group | 1.25 (1.06, 1.46) ** | 1.23 (1.00, 1.52) | 1.13 (0.89, 1.44) | 1.76 (1.18, 2.64) ** |

Hazard ratios were calculated by adjusting for age, sex, ethnicity, education level, Townsend deprivation index, smoking status, alcohol intake frequency, physical activity, diabetes duration, insulin use, body mass index, hypertension, triglyceride and high density lipoprotein cholesterol

SLD, steatotic liver disease

* *P* < 0.05, ** *P* < 0.01, *** *P* < 0.001

**Supplementary Figure 1** Study flow chart

**
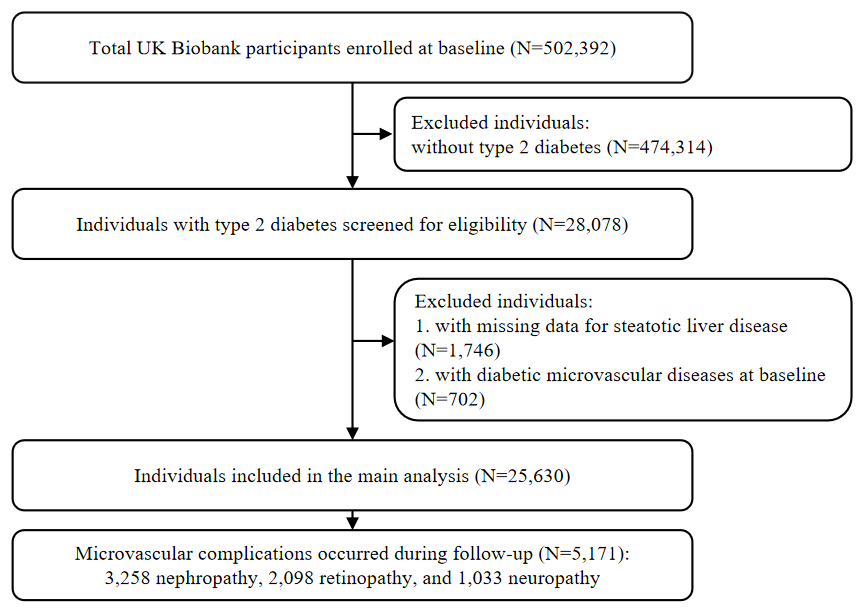
**

**Supplementary Figure 2** Cumulative incidence of diabetic microvascular complications


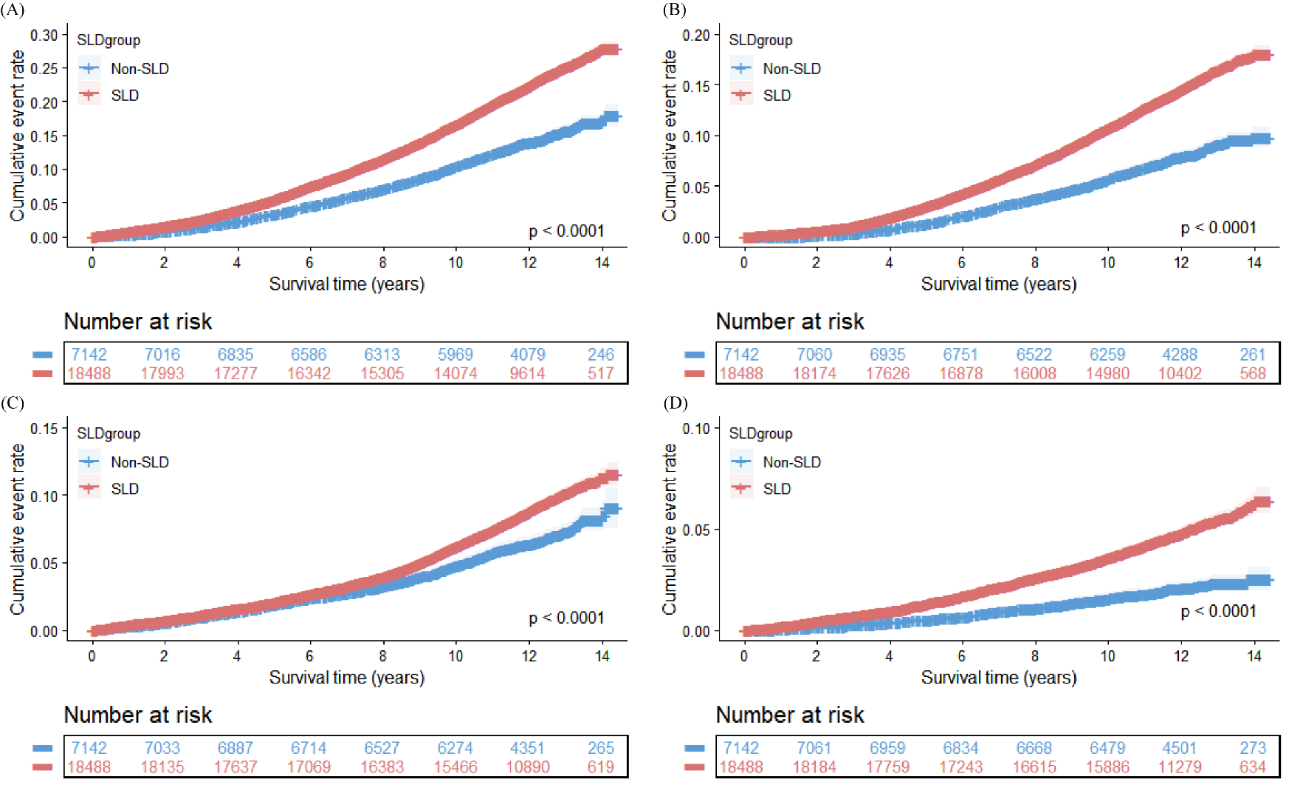


(A) Total microvascular complications, (B) Nephropathy, (C) Retinopathy, (D) Neuropathy

SLD, steatotic liver disease
